# Crystal structure and Hirshfeld surface analysis of 3-(2,6-di­chloro­phen­yl)-1,5-bis­(thio­phen-2-yl)pentane-1,5-dione

**DOI:** 10.1107/S2056989026010133

**Published:** 2026-10-02

**Authors:** Atash V. Gurbanov, Mehmet Akkurt, Gizachew Mulugeta Manahelohe

**Affiliations:** a Excellence Center, Baku State University, Z. Khalilov Str. 33, AZ 1148, Baku, Azerbaijan; b Department of Physics, Faculty of Sciences, Erciyes University, 38039 Kayseri, Türkiye; c Department of Chemistry, University of Gondar, PO Box 196, Gondar, Ethiopia; Universidad Nacional Autónoma de México, México

**Keywords:** crystal structure, disorder, thio­phene ring, non-covalent inter­actions, Hirshfeld surface analysis

## Abstract

In the crystal of the title compound, C_19_H_14_Cl_2_O_2_S_2_, mol­ecules are linked by inter­molecular C—H⋯O hydrogen bonds, forming a square-ring around a fourfold rotation axis parallel to the *c*-axis. In addition, C—H⋯π and π–π inter­actions [centroid-to-centroid distances = 3.759 (3) and 3.760 (3) Å] connect mol­ecules, forming a three-dimensional network. Two thio­phene rings are disordered with a 180° rotation over two positions; the first in a 0.686 (2)(3):0.314 (2) ratio and the second in a 0.787 (2):0.213 (2) ratio. The major contributions to the Hirshfeld surface are from H⋯H (32.4%), C⋯H/H⋯C (25.6%), Cl⋯H/H⋯Cl (17.2%) and O⋯H/H⋯O (14.4%) contacts.

## Abstract 2

In the crystal of the title compound, mol­ecules are linked by inter­molecular C—H⋯O hydrogen bonds, forming a square-ring around a fourfold rotation axis parallel to the *c*-axis. In addition, C—H⋯π and π-π- inter­actions connect mol­ecules, forming a three-dimensional network.

## 1. Chemical context

Thio­phenes with a five-membered planar ring are a family of sulfur-containing chemical compounds that are highly important in synthesis, crystal engineering, medicine and materials science (Nuralieva *et al.*, 2025 ▸, 2026 ▸; Torambetov *et al.*, 2026 ▸; Peng *et al.*, 2024 ▸). Selective functionalization of a thio­phene ring in homogeneous or heterogeneous catalytic systems (Makhmudova *et al.*, 2022 ▸; Mahmudov *et al.*, 2023 ▸) can be a powerful synthetic strategy towards new materials. In fact, substituted thio­phene drugs have been approved by the Food and Drug Administration (FDA), such as Cefoxitin, Raloxifene and Suprofen (Schweizer *et al.*, 2011 ▸), and these derivatives were used as multidentate ligands in coordination chemistry (Javadzade *et al.*, 2026 ▸; Mahmudov *et al.*, 2021 ▸). Halogenated derivatives of thio­phene are essential building blocks for synthesizing advanced functional materials like catalysts, organic solar cells, sensitizers and drugs (Guseinov *et al.*, 2025 ▸; Huseynov *et al.*, 2021 ▸; Mamedov *et al.*, 2024 ▸). Similarly to the fluorine analogue (Gurbanov *et al.*, 2025 ▸), attachment of a chlorine atom-containing residue to the thio­phene can lead to a new type of inter­molecular inter­actions. Thus, in the current work, we have synthesized the new title thio­phene derivative, (I), which presents multiple inter­molecular non-covalent inter­actions in the crystal packing.
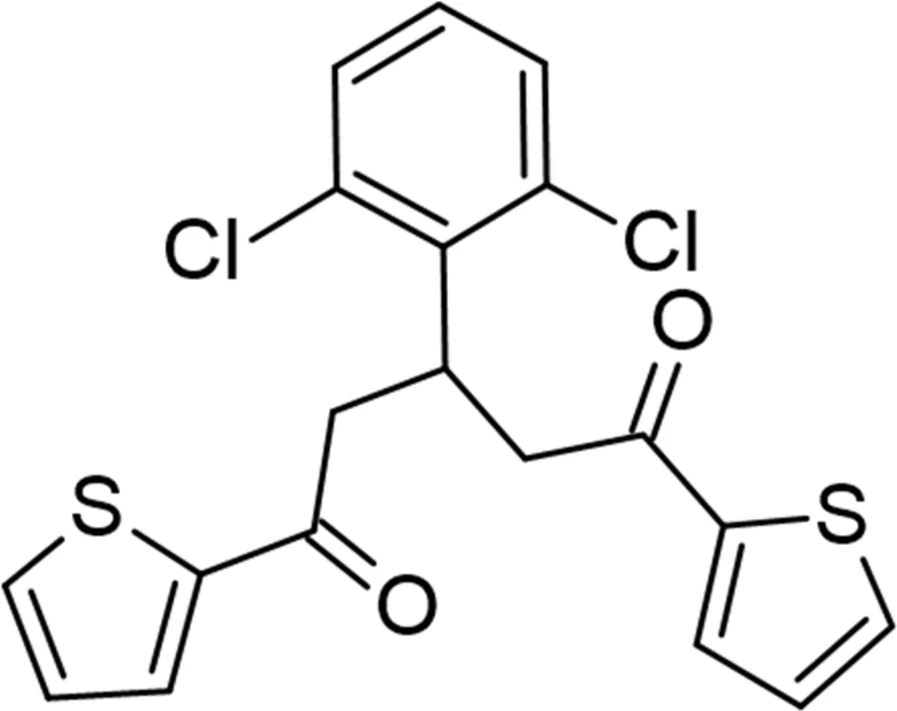


## 2. Structural commentary

The intra­molecular C10—H10*A*⋯Cl1 contact forms an *S*(6) ring motif (Bernstein *et al.*, 1995 ▸) with atoms C10, C9, C14, and C15, the intra­molecular C9—H9⋯Cl2 contact forms a *S*(5) ring motif with atoms C9, C14, and C19, the intra­molecular C12—H12*A*⋯Cl1 contact forms an *S*(6) ring motif with atoms C12, C9, C14, and C15, and the intra­molecular C9—H9⋯O1 contact forms an *S*(5) ring motif with atoms C9, C10, and C11 (Fig. 1 ▸, Table 1 ▸). The planes described by the di­chloro­phenyl (C14–C19) and the thio­phene rings (S1/C1–C4 and S2/C5–C8) are rotated by 45.5 (3) and 82.8 (2)°, respectively. The angle between these two thio­phene rings is 48.5 (3)°. The C14—C9—C10—C11, C9—C10—C11—C1, C9—C10—C11—O1, C14—C9—C12—C13, C9—C12—C13—O2 and C9—C12—C13—C5 torsion angles are 154.89 (12), 169.19 (12), −12.0 (2), −55.14 (16), −38.13 (19) and 143.98 (13)°, respectively. These inter­actions maintain the mol­ecular conformation. The geometric properties of the title compound are normal and consistent with those of similar compounds listed in the *Database survey* section.

## 3. Supra­molecular features and Hirshfeld surface analysis

In the crystal, mol­ecules are connected by C—H⋯O hydrogen bonds, which involve atoms O2, C1, C2 ,C9, C11, C10, C12, and C13, forming a square-ring around a fourfold rotation axis with graph set 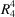
(40) (Fig. 2 ▸*a*,*b*,*c*; Table 1 ▸). Furthermore, C—H⋯π and π–π inter­actions [*Cg*1⋯*Cg*5^*b*^ = 3.759 (3) Å, slippage = 1.317 Å and *Cg*5⋯*Cg*1^*c*^ = 3.760 (3) Å, slippage = 1.572 Å; symmetry codes (*b*) *y*, 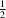 − *x*, 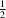 − *z*; (*c*) 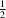 − *y*, *x*, 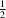 − *z*; *Cg*1 and *Cg*5 are the centroids of the (S1/C1—C4) thio­phene and (C14—C19) di­chloro­phenyl rings, respectively] connect mol­ecules, forming a three-dimensional network (Fig. 3 ▸*a*,*b*,*c*; Table 1 ▸).

A Hirshfeld surface analysis was performed with *CrystalExplorer* (Spackman *et al.*, 2021 ▸) to explore the inter­molecular inter­actions (Tables 1 ▸ and 2 ▸). In this analysis, the major and minor components of the disordered thio­phene rings in the refined CIF were considered together in the calculations. The Hirshfeld surface was mapped over *d*_norm_ in the range of −0.1652 to +1.4465 a.u. The red and blue areas of the Hirshfeld surface plotted over the *d*_norm_ (Fig. 4 ▸) indicate connections that are shorter or longer than the van der Waals radii, respectively, whereas the white surface shows contacts that are equal to the sum of the van der Waals radii. The most important inter­actions are H⋯H, C⋯H, Cl⋯H and O⋯H inter­actions (Tables 1 ▸ and 2 ▸).

Two-dimensional fingerprint plots together with their percentage contributions are shown in Fig. 5 ▸. The crystal packing is dominated by H⋯H contacts, which represent van der Waals inter­actions (32.4% of the entire surface), followed by C⋯H/H⋯C (25.6%), Cl⋯H/H⋯Cl (17.2%) and O⋯H/H⋯O (14.4%) inter­actions. Smaller contributions are observed for the S⋯H/H⋯S (3.5%), C⋯C (3.4%), Cl⋯Cl (1.7%), S⋯C/C⋯S (0.7%), S⋯O/O⋯S (0.5%), Cl⋯S/S⋯Cl (0.4%), O⋯O (0.1%), S⋯S (0.1%), O⋯C/C⋯O (0.1%) and Cl⋯C/C⋯Cl (0.1%) contacts.

## 4. Database survey

A search of the Cambridge Structural Database (CSD, Version 6.00, update of April 2025; Groom *et al.*, 2016 ▸) for closely related 3-aryl-1,5-bis(thiophen-2-yl)pentane-1,5-dione structures found 3-(2-chloro-6-fluoro­phen­yl)-1,5-bis­(thio­phen-2-yl)pentane-1,5-dione (AQILEV: Gurbanov *et al.*, 2025 ▸), 1,5-bis­(3-bromo­thien-2-yl)-3-(2,3,5-tri­chloro­phen­yl)pentane-1,5-dione (MIGVEE: Butcher *et al.*, 2007 ▸), 1,5-bis­(3-bromo­thien-2-yl)-3-(3-nitro­phen­yl)pentane-1,5-dione (WEP­JOR: Yathirajan *et al.*, 2006 ▸), 3-[4-(di­methyl­amino)­phen­yl]-1,5-bis­(thio­phen-2-yl)pentane-1,5-dione (XUJRAZ: Sharmoukh *et al.*, 2025 ▸) and 1,5-bis­(thio­phen-2-yl)-3-(2,4,5-tri­meth­oxy­phen­yl)pentane-1,5-dione (OCAXAT: Fun *et al.*, 2011 ▸).

The angles between the plane of the benzene ring and those of the two thio­phene rings are 40.9 (1) and 51.6 (1)° for AQILEV, 89.7 (5) and 63.7 (1)° for MIGVEE, 84.9 (2) and 68.8 (2)° for WEPJOR, 75.27 (5) and 83.8 (2)° for XUJRAZ, and 41.51 (6) and 25.83 (6)° for OCAXAT.

In the crystal of AQILEV, the mol­ecules are linked into [010] chains by C—H⋯O hydrogen bonds, forming a *C*(6) motif; weak C—H⋯F and C—H⋯Cl inter­actions link these chains into sheets parallel to the (100) plane. In MIGVEE, the mol­ecules are linked by C—Br⋯π and C—Cl⋯π inter­actions and there are no classical hydrogen bonds. In WEPJOR, the mol­ecules form layers parallel to (200) through C—H⋯O hydrogen bonds. C—H⋯π, C—Br⋯π, and C—O⋯π inter­actions formed between the layers strengthen the mol­ecular packing. In the crystal of XUJRAZ, C—H⋯O hydrogen bonds form chains of mol­ecules extending along the *c*-axis direction. These are linked by C—H⋯S hydrogen bonds and C—H⋯π inter­actions into corrugated layers parallel to the *bc* plane. In OCAXAT, mol­ecules are linked into a three-dimensional network by weak C—H⋯O and C—H⋯π inter­actions, and a π–π stacking with a centroid–centroid distance of 3.6527 (8) Å. The respective observed pattern in the crystal of the title compound is, hence, also notably uncommon.

## 5. Synthesis and crystallization

2,6-Di­chloro­benzaldehyde (10 mmol) was added to a solution of 1-(thio­phen-2-yl)ethan-1-one (20 mmol) in EtOH (60 ml). KOH pellets (25 mmol) were then added to the solution (Fig. 6 ▸). The solution was stirred at room temperature for 8 h. The off-white solid was collected by filtration and washed with EtOH (3 × 10 ml). Recrystallization from CHCl_3_–MeOH afforded a white crystalline solid of the title compound (yield, 64%). Analysis calculated for C_19_H_14_Cl_2_O_2_S_2_: C 55.75, H 3.45. Found: C 55.72, H 3.43. ^1^H NMR (400 MHz, CDCl_3_): δ = 4.12–4.16 (1H, CH), 3.36-3.52 (4H, 2CH_2_), 7.14–8.26 (9H, Ar and thio­phen-2-yl) ppm. ^13^C NMR (100 MHz, CDCl_3_): δ = 196.4, 160.0, 142.2, 136.8, 135.8, 133.4, 132.2, 129.8, 128.0, 46.8, 34.9 ppm.

## 6. Refinement

Crystal data, data collection and structure refinement details are summarized in Table 3 ▸. All H atoms were positioned geometrically (C—H = 0.95–1.00 Å) and refined as riding with *U*_iso_(H) = 1.2–1.5*U*_eq_(C). Two thio­phene rings (S1/C1–C4 and S2/C5–C8) are disordered with a 180° rotation over two positions; the first around the C1–C11 bond with a 0.686 (2)(3):0.314 (2) ratio and the second around the C5–C13 bond with a 0.787 (2):0.213 (2) ratio. The restraints on bond lengths and atomic displacement parameters on each pair of disorder fragments (SADI and SAME instructions of *SHELXL*) as well as the restraints of the atomic displacement parameters (SIMU/RIGU instructions of *SHELXL*) were applied for the atoms of the two disorder thio­phene rings (S1/C1–C4 and S2/C5–C8). Five outlier reflections (110, 020, 011, 021 and 121), affected by the incident beam-stop were omitted in the last cycles of the refinement.

## Supplementary Material

Crystal structure: contains datablock(s) I. DOI: 10.1107/S2056989026010133/jq2043sup1.cif

Structure factors: contains datablock(s) I. DOI: 10.1107/S2056989026010133/jq2043Isup2.hkl

Supporting information file. DOI: 10.1107/S2056989026010133/jq2043Isup3.cml

CCDC reference: 2591583

Additional supporting information:  crystallographic information; 3D view; checkCIF report

## Figures and Tables

**Figure 1:**
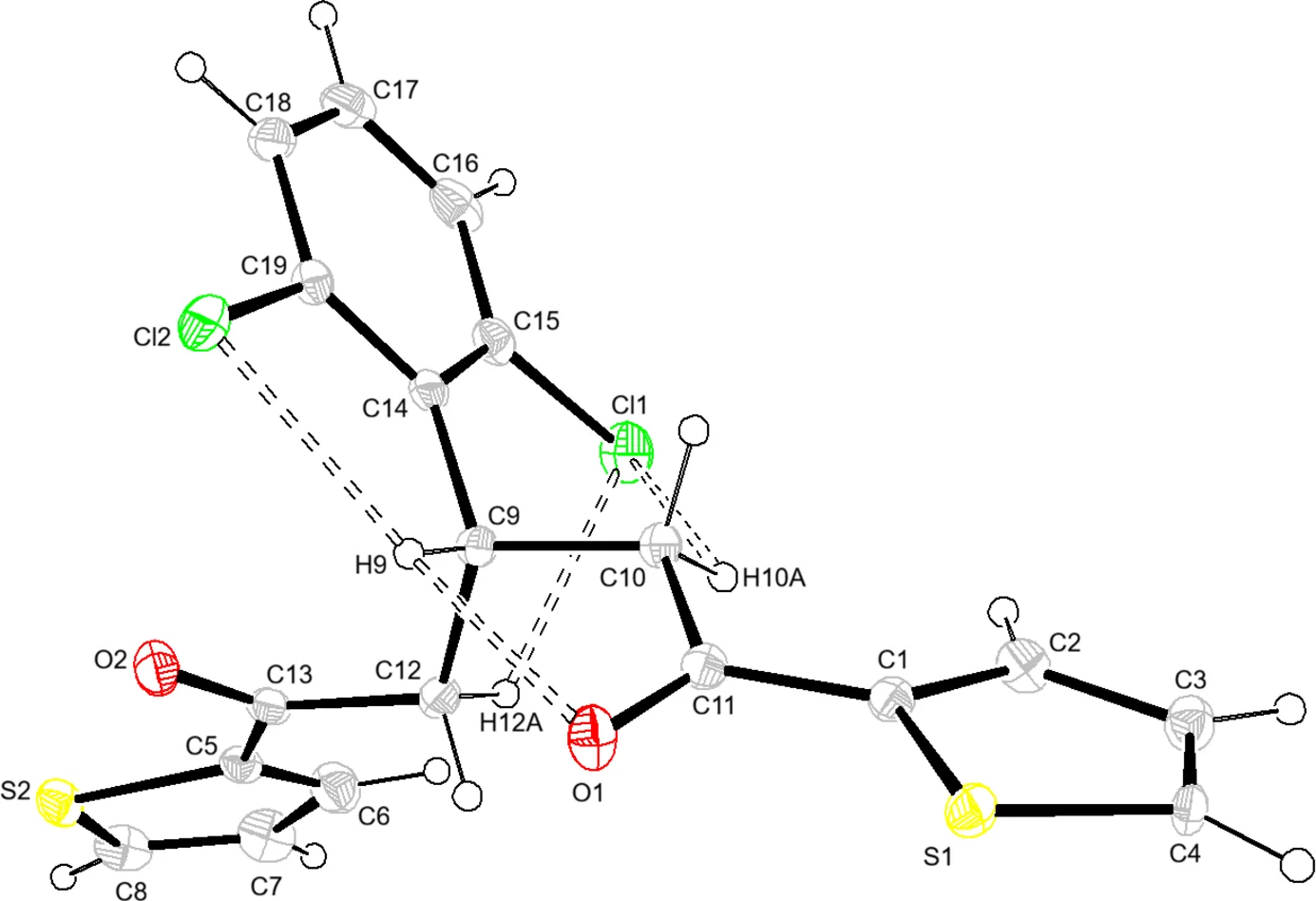
Mol­ecular structure showing the atomic labelling. Displacement ellipsoids are drawn at the 30% probability level. Minor disorder components have been omitted for clarity.

**Figure 2:**
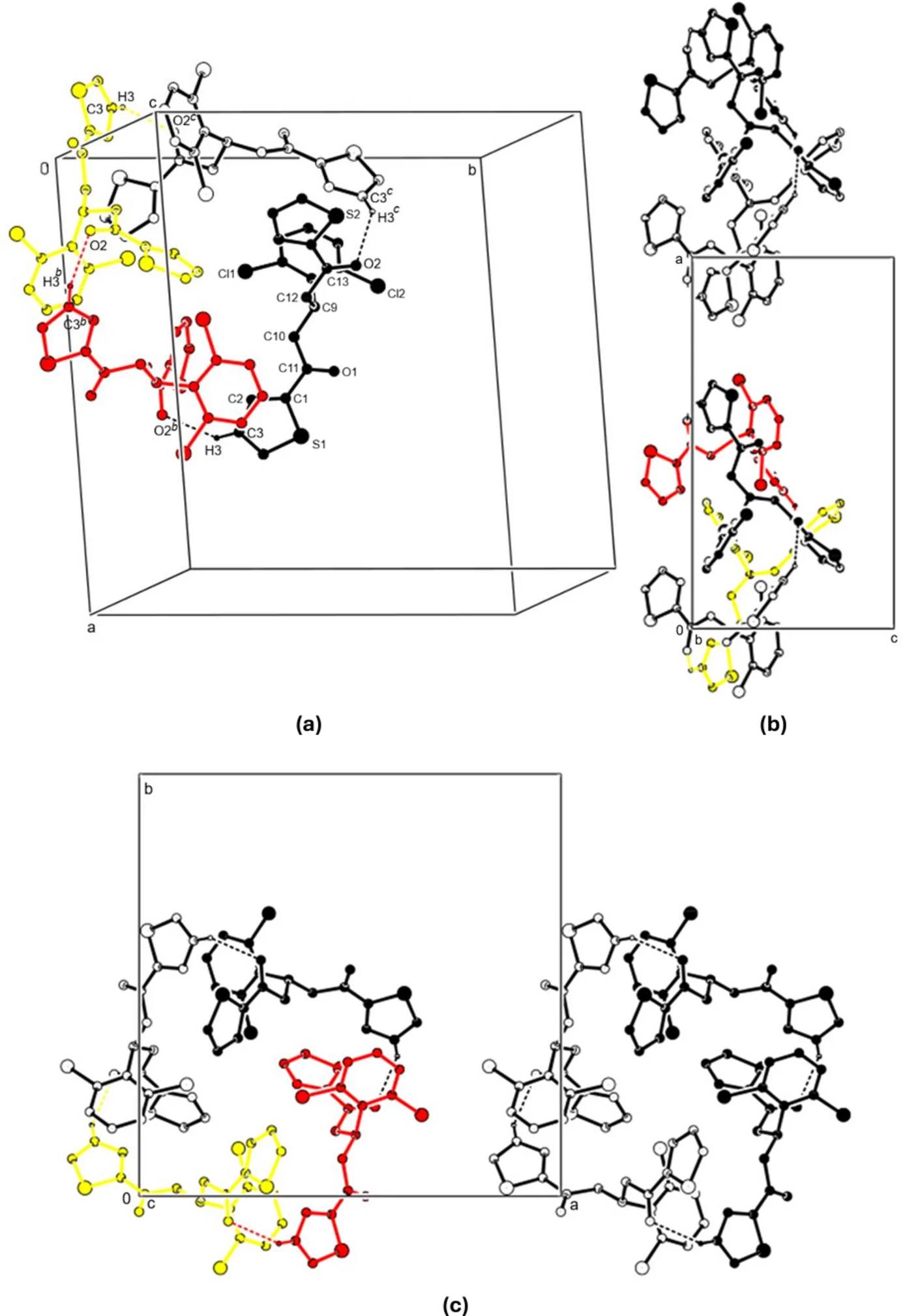
(*a*) A partial view of the C—H⋯O inter­actions in the unit cell. (*b*) A view down the *b* axis of the C—H⋯O inter­actions. (*c*) Crystal packing viewed along the *c* axis showing the C—H⋯O inter­actions (dashed lines) for the situation shown in Fig. 2 ▸*b*. Minor disorder components and hydrogen atoms not involved in hydrogen bonding have been omitted for clarity. Symmetry codes: (*b*) *y*, 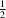 − *x*, 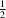 − *z*; (*c*) 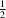 − *y*, *x*, 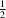 − *z*.

**Figure 3:**
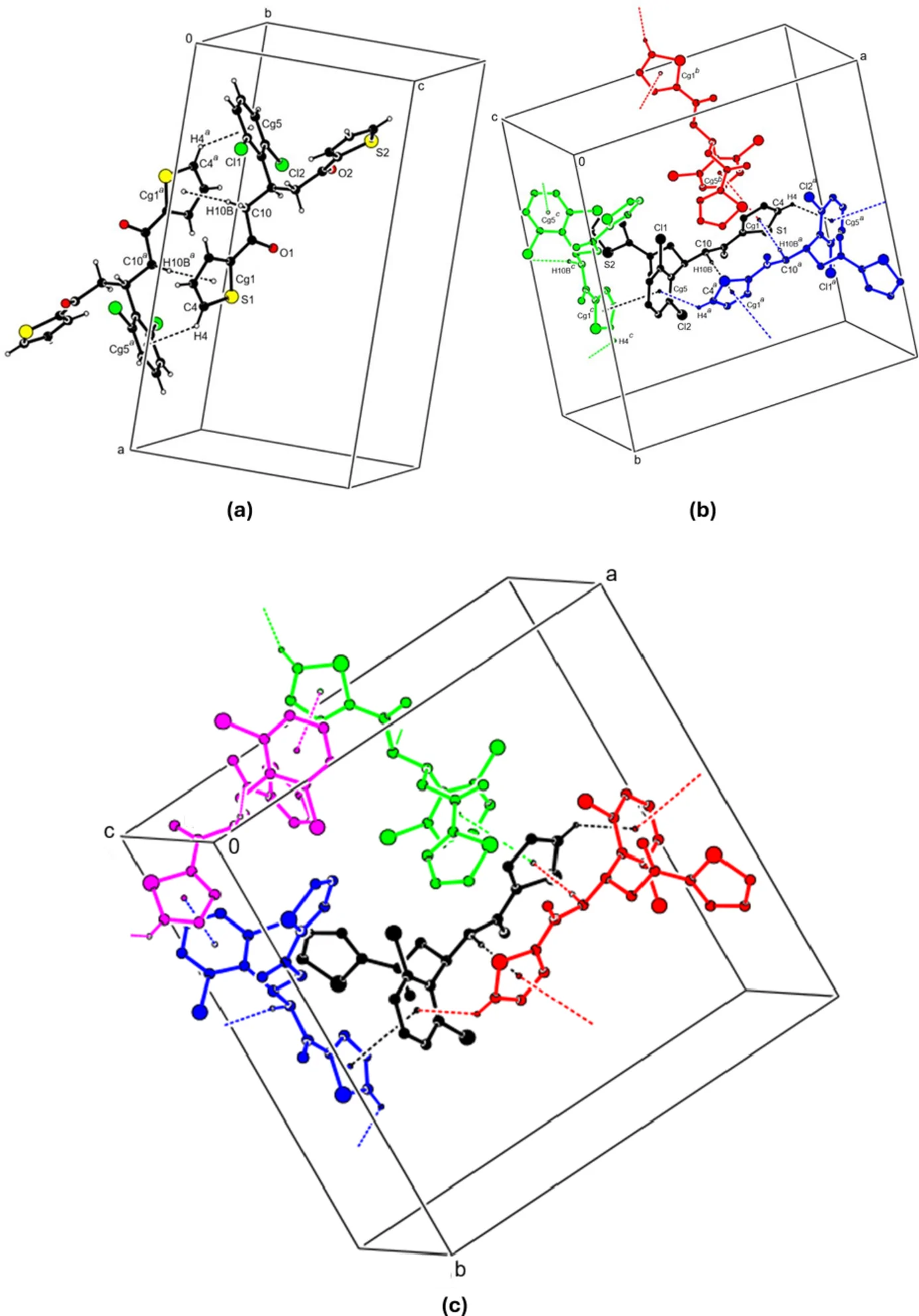
(*a*) A view of the C—H⋯π inter­actions between two adjacent mol­ecules in the unit cell (dashed lines). (*b*) A partial view of the C—H⋯π and π–π inter­actions (dashed lines) in the unit cell. (*c*) Partial view of C—H⋯π and π–π inter­actions and C—H⋯O hydrogen bonds (dashed lines) in the unit cell. For clarity, minor disorder components have been removed in all of them, and in (*b*) and (*c*) hydrogen atoms not involved in hydrogen bonding have been removed. Symmetry codes: (*a*) 1 − *x*, 1 − *y*, − *z*; (*b*) *y*, 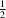 − *x*, 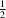 − *z*; (*c*) 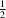 − *y*, *x*, 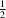 − *z*.

**Figure 4:**
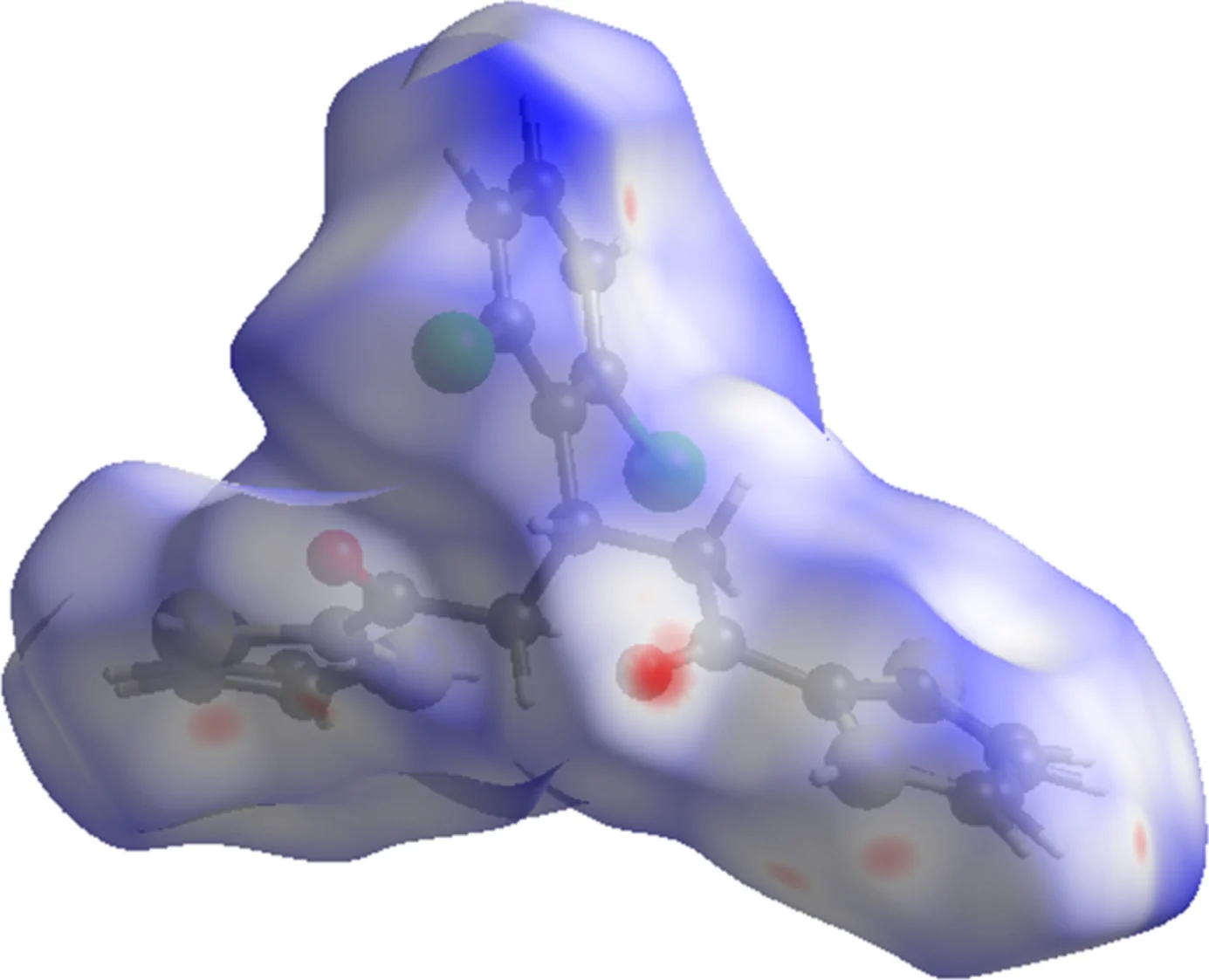
Three-dimensional Hirshfeld surface analysis mapped over *d*_norm_.

**Figure 5:**
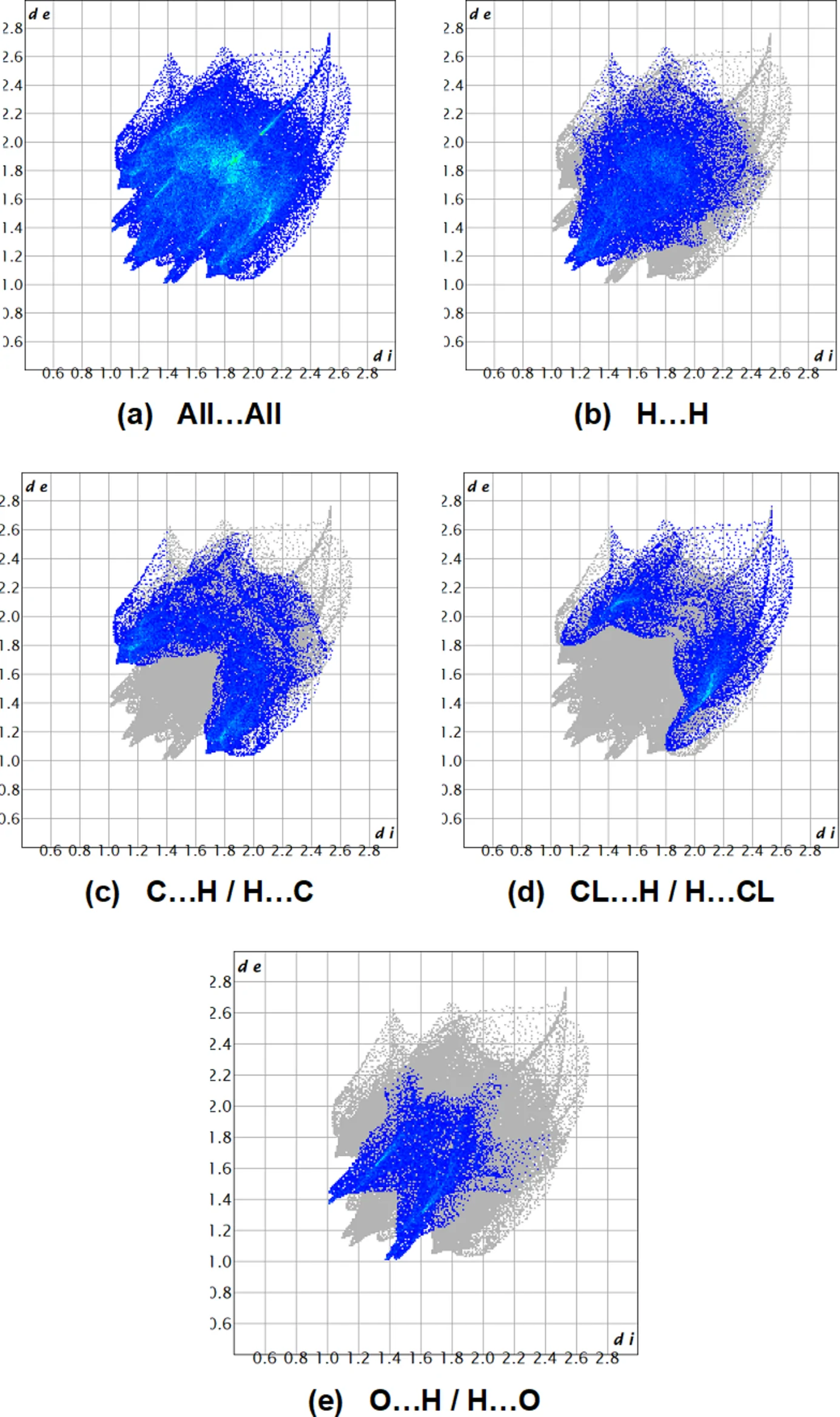
The two-dimensional fingerprint plots showing (*a*) all inter­actions, and those delineated into (*b*) H⋯H, (*c*) C⋯H/H⋯C, (*d*) Cl⋯H/H⋯Cl and (*e*) O⋯H/H⋯O inter­actions. The *d*_i_ and *d*_e_ values are the closest inter­nal and external distances (in Å) from given points on the Hirshfeld surface.

**Figure 6:**
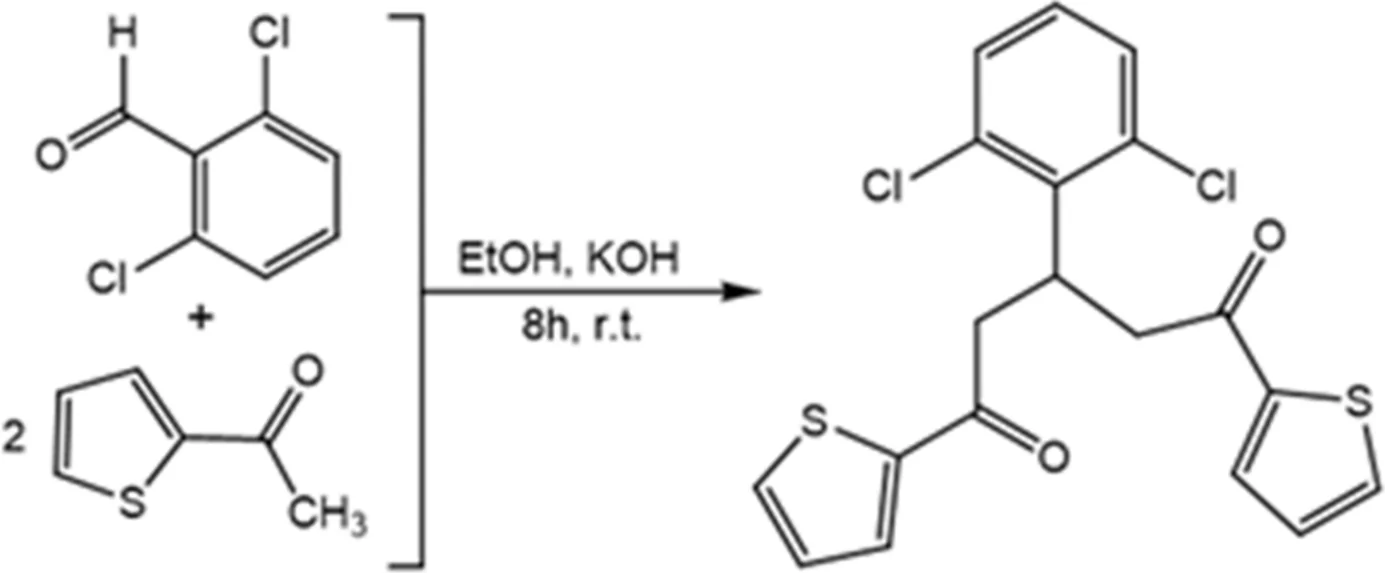
The reaction scheme of the title compound.

**Table 1:** Hydrogen-bond geometry (Å, °)

*D*—H⋯*A*	*D*—H	H⋯*A*	*D*⋯*A*	*D*—H⋯*A*
C3—H3⋯O2^i^	0.95	2.48	3.238 (8)	136
C9—H9⋯Cl2	1.00	2.47	3.0884 (18)	120
C9—H9⋯O1	1.00	2.44	2.819 (2)	102
C10—H10*A*⋯Cl1	0.99	2.75	3.3095 (19)	116
C12—H12*A*⋯Cl1	0.99	2.64	3.317 (2)	126
C4—H4⋯*Cg*5^ii^	0.95	2.83	3.431 (7)	122
C10—H10*B*⋯*Cg*1^ii^	0.99	2.72	3.704 (4)	171

Symmetry codes: (i) 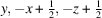; (ii) 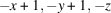.

**Table 2:** Summary of short inter­atomic contacts (Å)

Contact	Distance	Symmetry operation
*H3⋯O2	2.48	*y*, 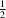 − *x*, 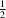 − *z*
*H4⋯C18	2.83	1 − *x*, 1 − *y*, −*z*
*H2*X*⋯*H8	2.42	1 − *y*, 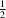 + *x*, − 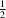 + *z*
*H2*X*⋯C13	2.94	1 − *x*, 1 − *y*, 1 − *z*
H9⋯H17	2.56	1 − *y*, 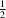 + *x*, 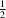 + *z*
*C7⋯*H7	2.93	*y*, 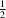 − *x*, 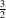 − *z*

The prefix * indicates an atom of a disordered component.

**Table 3:** Experimental details

Crystal data	
Chemical formula	C_19_H_14_Cl_2_O_2_S_2_
*M*_r_	409.32
Crystal system, space group	Tetragonal, *P*4_2_/*n*
Temperature (K)	150
*a*, *c* (Å)	18.853 (3), 10.235 (4)
*V* (Å^3^)	3637.9 (18)
*Z*	8
Radiation type	Mo *K*α
μ (mm^−1^)	0.60
Crystal size (mm)	0.36 × 0.23 × 0.23

Data collection	
Diffractometer	Bruker APEXII CCD
No. of measured, independent and observed [*I* > 2σ(*I*)] reflections	34717, 3693, 3195
*R*_int_	0.026
(sin θ/λ)_max_ (Å^−1^)	0.624

Refinement	
*R*[*F*^2^ > 2σ(*F*^2^)], *wR*(*F*^2^), *S*	0.027, 0.074, 1.04
No. of reflections	3693
No. of parameters	300
No. of restraints	608
H-atom treatment	H-atom parameters constrained
Δρ_max_, Δρ_min_ (e Å^−3^)	0.29, −0.20

Computer programs: *APEX2* and *SAINT* (Bruker, 2012 ▸), *SHELXT* (Sheldrick, 2015*a* ▸), *SHELXL* (Sheldrick, 2015*b* ▸), *ORTEP-3 for Windows* (Farrugia, 2012 ▸) and *PLATON* (Spek, 2020 ▸).

## supplementary crystallographic information

### 3-(2,6-Dichlorophenyl)-1,5-bis(thiophen-2-yl)pentane-1,5-dione Crystal data

**Table:**

C_19_H_14_Cl_2_O_2_S_2_	*D*_x_ = 1.495 Mg m^−^^3^
*M**_r_* = 409.32	Mo *K*α radiation, λ = 0.71073 Å
Tetragonal, *P*4_2_/*n*	Cell parameters from 9964 reflections
*a* = 18.853 (3) Å	θ = 2.5–26.4°
*c* = 10.235 (4) Å	µ = 0.60 mm^−^^1^
*V* = 3637.9 (18) Å^3^	*T* = 150 K
*Z* = 8	Plate, yellow
*F*(000) = 1680	0.36 × 0.23 × 0.23 mm

### 3-(2,6-Dichlorophenyl)-1,5-bis(thiophen-2-yl)pentane-1,5-dione Data collection

**Table:**

Bruker APEXII CCD diffractometer	*R*_int_ = 0.026
φ and ω scans	θ_max_ = 26.3°, θ_min_ = 3.1°
34717 measured reflections	*h* = −23→23
3693 independent reflections	*k* = −23→23
3195 reflections with *I* > 2σ(*I*)	*l* = −7→12

### 3-(2,6-Dichlorophenyl)-1,5-bis(thiophen-2-yl)pentane-1,5-dione Refinement

**Table:**

Refinement on *F*^2^	608 restraints
Least-squares matrix: full	Hydrogen site location: inferred from neighbouring sites
*R*[*F*^2^ > 2σ(*F*^2^)] = 0.027	H-atom parameters constrained
*wR*(*F*^2^) = 0.074	*w* = 1/[σ^2^(*F*_o_^2^) + (0.0383*P*)^2^ + 1.3968*P*] where *P* = (*F*_o_^2^ + 2*F*_c_^2^)/3
*S* = 1.04	(Δ/σ)_max_ = 0.001
3693 reflections	Δρ_max_ = 0.29 e Å^−^^3^
300 parameters	Δρ_min_ = −0.20 e Å^−^^3^

### 3-(2,6-Dichlorophenyl)-1,5-bis(thiophen-2-yl)pentane-1,5-dione Special details

**Table:**

Geometry. All esds (except the esd in the dihedral angle between two l.s. planes) are estimated using the full covariance matrix. The cell esds are taken into account individually in the estimation of esds in distances, angles and torsion angles; correlations between esds in cell parameters are only used when they are defined by crystal symmetry. An approximate (isotropic) treatment of cell esds is used for estimating esds involving l.s. planes.

### 3-(2,6-Dichlorophenyl)-1,5-bis(thiophen-2-yl)pentane-1,5-dione Fractional atomic coordinates and isotropic or equivalent isotropic displacement parameters (Å^2^)

**Table:**

	*x*	*y*	*z*	*U*_iso_*/*U*_eq_	Occ. (<1)
C1	0.54327 (7)	0.45619 (7)	0.17318 (14)	0.0213 (3)	
C2	0.5388 (3)	0.3969 (4)	0.0932 (9)	0.0290 (13)	0.686 (2)
H2	0.495095	0.375484	0.069437	0.035*	0.686 (2)
C3	0.6049 (2)	0.3716 (4)	0.0506 (10)	0.0286 (9)	0.686 (2)
H3	0.611278	0.331904	−0.005434	0.034*	0.686 (2)
C4	0.6592 (2)	0.4118 (4)	0.1008 (8)	0.0250 (9)	0.686 (2)
H4	0.708035	0.402763	0.084738	0.030*	0.686 (2)
S1	0.62866 (5)	0.48026 (8)	0.19615 (17)	0.0256 (2)	0.686 (2)
C2X	0.6159 (4)	0.4696 (6)	0.1882 (14)	0.0295 (19)	0.314 (2)
H2X	0.634291	0.506938	0.240594	0.035*	0.314 (2)
C3X	0.6587 (4)	0.4224 (8)	0.1180 (17)	0.0252 (18)	0.314 (2)
H3X	0.708972	0.424644	0.114403	0.030*	0.314 (2)
C4X	0.6185 (4)	0.3724 (9)	0.055 (2)	0.0264 (19)	0.314 (2)
H4X	0.637537	0.334632	0.004448	0.032*	0.314 (2)
S1X	0.52868 (18)	0.3865 (2)	0.0791 (5)	0.0254 (6)	0.314 (2)
C5	0.24771 (7)	0.44532 (8)	0.57830 (13)	0.0235 (3)	
C6	0.2346 (4)	0.3742 (2)	0.5597 (6)	0.0396 (11)	0.787 (2)
H6	0.256125	0.347344	0.491950	0.048*	0.787 (2)
C7	0.1870 (3)	0.3451 (2)	0.6494 (6)	0.0408 (10)	0.787 (2)
H7	0.173350	0.296622	0.650717	0.049*	0.787 (2)
C8	0.1625 (4)	0.3950 (2)	0.7348 (7)	0.0349 (9)	0.787 (2)
H8	0.129320	0.385120	0.802326	0.042*	0.787 (2)
S2	0.19795 (5)	0.47731 (4)	0.70732 (9)	0.0295 (2)	0.787 (2)
C6X	0.2054 (8)	0.4615 (5)	0.6847 (12)	0.036 (2)	0.213 (2)
H6X	0.204915	0.506882	0.724860	0.043*	0.213 (2)
C7X	0.1634 (15)	0.4052 (8)	0.728 (2)	0.035 (2)	0.213 (2)
H7X	0.132681	0.407117	0.801252	0.043*	0.213 (2)
C8X	0.1725 (10)	0.3468 (7)	0.650 (2)	0.033 (2)	0.213 (2)
H8X	0.145785	0.304283	0.657880	0.040*	0.213 (2)
S2X	0.2382 (4)	0.3595 (2)	0.5347 (6)	0.0376 (10)	0.213 (2)
C9	0.35448 (7)	0.51320 (7)	0.28580 (13)	0.0212 (3)	
H9	0.373277	0.559825	0.316950	0.025*	
C10	0.41125 (7)	0.48186 (8)	0.19393 (14)	0.0244 (3)	
H10A	0.403534	0.430095	0.186030	0.029*	
H10B	0.405555	0.502934	0.105964	0.029*	
C11	0.48646 (7)	0.49511 (7)	0.24127 (14)	0.0220 (3)	
C12	0.34579 (7)	0.46603 (8)	0.40816 (14)	0.0245 (3)	
H12A	0.330170	0.418175	0.380605	0.029*	
H12B	0.392426	0.461014	0.451647	0.029*	
C13	0.29274 (7)	0.49555 (7)	0.50549 (13)	0.0226 (3)	
C14	0.28523 (7)	0.52898 (8)	0.21384 (13)	0.0218 (3)	
C15	0.24264 (8)	0.47786 (8)	0.15209 (15)	0.0277 (3)	
C16	0.18201 (8)	0.49449 (10)	0.08089 (16)	0.0355 (4)	
H16	0.155454	0.457985	0.039652	0.043*	
C17	0.16059 (8)	0.56445 (10)	0.07042 (16)	0.0376 (4)	
H17	0.119155	0.576165	0.022198	0.045*	
C18	0.19967 (8)	0.61723 (10)	0.13036 (15)	0.0332 (4)	
H18	0.185163	0.665370	0.124235	0.040*	
C19	0.26051 (8)	0.59915 (8)	0.19980 (14)	0.0259 (3)	
O1	0.50021 (5)	0.53666 (6)	0.32983 (11)	0.0315 (3)	
O2	0.28861 (6)	0.55928 (5)	0.52630 (10)	0.0312 (2)	
Cl1	0.26457 (2)	0.38719 (2)	0.16007 (5)	0.04004 (12)	
Cl2	0.30752 (2)	0.66987 (2)	0.26885 (4)	0.03587 (11)	

### 3-(2,6-Dichlorophenyl)-1,5-bis(thiophen-2-yl)pentane-1,5-dione Atomic displacement parameters (Å^2^)

**Table:**

	*U*^11^	*U*^22^	*U*^33^	*U*^12^	*U*^13^	*U*^23^
C1	0.0217 (7)	0.0211 (7)	0.0212 (7)	0.0004 (5)	0.0025 (5)	0.0041 (5)
C2	0.027 (2)	0.031 (3)	0.028 (2)	−0.0061 (16)	0.0004 (16)	−0.0019 (16)
C3	0.0314 (17)	0.0242 (15)	0.0302 (18)	0.0018 (17)	0.005 (2)	0.0001 (13)
C4	0.0243 (16)	0.027 (2)	0.023 (2)	0.0107 (13)	0.0073 (13)	0.0000 (13)
S1	0.0162 (3)	0.0319 (5)	0.0287 (4)	−0.0003 (3)	−0.0004 (3)	−0.0022 (3)
C2X	0.033 (3)	0.029 (4)	0.027 (3)	−0.003 (3)	−0.002 (3)	0.005 (3)
C3X	0.024 (3)	0.026 (4)	0.026 (4)	−0.004 (2)	−0.003 (3)	0.005 (3)
C4X	0.021 (3)	0.028 (3)	0.030 (4)	0.007 (3)	0.012 (3)	−0.002 (3)
S1X	0.0266 (11)	0.0230 (10)	0.0266 (11)	−0.0035 (8)	0.0053 (8)	−0.0080 (7)
C5	0.0199 (7)	0.0310 (7)	0.0197 (7)	−0.0003 (5)	−0.0027 (5)	0.0011 (6)
C6	0.0379 (17)	0.048 (2)	0.033 (2)	0.0014 (17)	0.0022 (15)	−0.0013 (15)
C7	0.035 (2)	0.0426 (14)	0.0445 (17)	−0.0132 (13)	−0.0007 (15)	0.0033 (13)
C8	0.0265 (14)	0.0465 (18)	0.0317 (16)	−0.0119 (16)	0.0020 (12)	0.0101 (15)
S2	0.0254 (3)	0.0393 (4)	0.0238 (4)	−0.0022 (3)	0.0062 (2)	0.0010 (3)
C6X	0.035 (4)	0.046 (4)	0.027 (4)	0.004 (3)	−0.005 (3)	0.001 (3)
C7X	0.030 (4)	0.050 (4)	0.027 (4)	0.006 (4)	0.003 (4)	0.002 (4)
C8X	0.025 (5)	0.045 (4)	0.030 (4)	−0.010 (3)	0.004 (4)	0.006 (3)
S2X	0.0422 (16)	0.0377 (17)	0.033 (2)	−0.0115 (14)	0.0100 (13)	0.0022 (14)
C9	0.0193 (7)	0.0245 (7)	0.0198 (7)	0.0027 (5)	0.0013 (5)	0.0008 (6)
C10	0.0223 (7)	0.0292 (7)	0.0218 (7)	0.0038 (6)	0.0024 (6)	0.0007 (6)
C11	0.0243 (7)	0.0200 (7)	0.0218 (7)	0.0002 (5)	0.0040 (6)	0.0048 (6)
C12	0.0239 (7)	0.0263 (7)	0.0234 (7)	0.0054 (6)	0.0027 (6)	0.0036 (6)
C13	0.0212 (7)	0.0289 (7)	0.0176 (7)	0.0014 (6)	−0.0026 (5)	0.0015 (6)
C14	0.0199 (7)	0.0286 (7)	0.0169 (6)	0.0028 (6)	0.0038 (5)	0.0010 (6)
C15	0.0258 (7)	0.0347 (8)	0.0227 (7)	−0.0008 (6)	0.0053 (6)	−0.0018 (6)
C16	0.0231 (8)	0.0603 (11)	0.0230 (8)	−0.0085 (7)	0.0019 (6)	−0.0035 (8)
C17	0.0202 (7)	0.0684 (12)	0.0240 (8)	0.0082 (8)	0.0010 (6)	0.0069 (8)
C18	0.0276 (8)	0.0478 (10)	0.0242 (8)	0.0153 (7)	0.0052 (6)	0.0068 (7)
C19	0.0258 (7)	0.0316 (8)	0.0202 (7)	0.0054 (6)	0.0042 (6)	0.0003 (6)
O1	0.0285 (5)	0.0316 (6)	0.0345 (6)	−0.0019 (4)	0.0045 (5)	−0.0096 (5)
O2	0.0393 (6)	0.0266 (6)	0.0276 (6)	0.0004 (5)	0.0070 (5)	−0.0022 (4)
Cl1	0.0426 (2)	0.0314 (2)	0.0462 (3)	−0.00663 (16)	0.00020 (19)	−0.00862 (18)
Cl2	0.0449 (2)	0.02447 (19)	0.0383 (2)	0.00821 (16)	−0.00231 (18)	−0.00167 (16)

### 3-(2,6-Dichlorophenyl)-1,5-bis(thiophen-2-yl)pentane-1,5-dione Geometric parameters (Å, º)

**Table:**

C1—C2	1.388 (5)	C6X—H6X	0.9500
C1—C2X	1.402 (7)	C7X—C8X	1.373 (7)
C1—C11	1.4734 (19)	C7X—H7X	0.9500
C1—S1X	1.652 (3)	C8X—S2X	1.725 (8)
C1—S1	1.6891 (16)	C8X—H8X	0.9500
C2—C3	1.404 (7)	C9—C14	1.5283 (19)
C2—H2	0.9500	C9—C10	1.5423 (19)
C3—C4	1.373 (4)	C9—C12	1.545 (2)
C3—H3	0.9500	C9—H9	1.0000
C4—S1	1.718 (4)	C10—C11	1.519 (2)
C4—H4	0.9500	C10—H10A	0.9900
C2X—C3X	1.400 (9)	C10—H10B	0.9900
C2X—H2X	0.9500	C11—O1	1.2257 (18)
C3X—C4X	1.370 (6)	C12—C13	1.517 (2)
C3X—H3X	0.9500	C12—H12A	0.9900
C4X—S1X	1.732 (7)	C12—H12B	0.9900
C4X—H4X	0.9500	C13—O2	1.2228 (17)
C5—C6	1.376 (5)	C14—C15	1.405 (2)
C5—C6X	1.384 (7)	C14—C19	1.410 (2)
C5—C13	1.4740 (19)	C15—C16	1.391 (2)
C5—S2X	1.688 (4)	C15—Cl1	1.7605 (16)
C5—S2	1.7285 (16)	C16—C17	1.384 (3)
C6—C7	1.396 (6)	C16—H16	0.9500
C6—H6	0.9500	C17—C18	1.382 (3)
C7—C8	1.365 (4)	C17—H17	0.9500
C7—H7	0.9500	C18—C19	1.392 (2)
C8—S2	1.713 (3)	C18—H18	0.9500
C8—H8	0.9500	C19—Cl2	1.7500 (16)
C6X—C7X	1.394 (9)		
			
C2—C1—C11	129.5 (3)	C6X—C7X—H7X	124.6
C2X—C1—C11	124.7 (4)	C7X—C8X—S2X	112.1 (6)
C2X—C1—S1X	111.7 (3)	C7X—C8X—H8X	124.0
C11—C1—S1X	123.42 (15)	S2X—C8X—H8X	124.0
C2—C1—S1	110.8 (3)	C5—S2X—C8X	91.7 (4)
C11—C1—S1	119.55 (11)	C14—C9—C10	111.94 (11)
C1—C2—C3	113.8 (4)	C14—C9—C12	114.34 (11)
C1—C2—H2	123.1	C10—C9—C12	110.33 (11)
C3—C2—H2	123.1	C14—C9—H9	106.6
C4—C3—C2	110.9 (4)	C10—C9—H9	106.6
C4—C3—H3	124.5	C12—C9—H9	106.6
C2—C3—H3	124.5	C11—C10—C9	112.97 (12)
C3—C4—S1	112.2 (3)	C11—C10—H10A	109.0
C3—C4—H4	123.9	C9—C10—H10A	109.0
S1—C4—H4	123.9	C11—C10—H10B	109.0
C1—S1—C4	92.23 (16)	C9—C10—H10B	109.0
C3X—C2X—C1	113.0 (5)	H10A—C10—H10B	107.8
C3X—C2X—H2X	123.5	O1—C11—C1	120.95 (13)
C1—C2X—H2X	123.5	O1—C11—C10	122.57 (13)
C4X—C3X—C2X	111.2 (6)	C1—C11—C10	116.47 (12)
C4X—C3X—H3X	124.4	C13—C12—C9	113.02 (11)
C2X—C3X—H3X	124.4	C13—C12—H12A	109.0
C3X—C4X—S1X	111.6 (6)	C9—C12—H12A	109.0
C3X—C4X—H4X	124.2	C13—C12—H12B	109.0
S1X—C4X—H4X	124.2	C9—C12—H12B	109.0
C1—S1X—C4X	92.4 (3)	H12A—C12—H12B	107.8
C6—C5—C13	131.2 (2)	O2—C13—C5	120.43 (13)
C6X—C5—C13	126.1 (4)	O2—C13—C12	121.12 (13)
C6X—C5—S2X	110.9 (4)	C5—C13—C12	118.42 (12)
C13—C5—S2X	122.91 (19)	C15—C14—C19	114.14 (13)
C6—C5—S2	110.4 (2)	C15—C14—C9	124.86 (13)
C13—C5—S2	118.34 (11)	C19—C14—C9	120.94 (13)
C5—C6—C7	114.0 (4)	C16—C15—C14	123.41 (15)
C5—C6—H6	123.0	C16—C15—Cl1	115.85 (12)
C7—C6—H6	123.0	C14—C15—Cl1	120.74 (11)
C8—C7—C6	111.6 (3)	C17—C16—C15	119.69 (15)
C8—C7—H7	124.2	C17—C16—H16	120.2
C6—C7—H7	124.2	C15—C16—H16	120.2
C7—C8—S2	112.7 (3)	C18—C17—C16	119.76 (14)
C7—C8—H8	123.6	C18—C17—H17	120.1
S2—C8—H8	123.6	C16—C17—H17	120.1
C8—S2—C5	91.23 (13)	C17—C18—C19	119.33 (16)
C5—C6X—C7X	114.1 (6)	C17—C18—H18	120.3
C5—C6X—H6X	122.9	C19—C18—H18	120.3
C7X—C6X—H6X	122.9	C18—C19—C14	123.66 (15)
C8X—C7X—C6X	110.8 (7)	C18—C19—Cl2	115.91 (12)
C8X—C7X—H7X	124.6	C14—C19—Cl2	120.42 (11)
			
C11—C1—C2—C3	−176.6 (6)	C2X—C1—C11—C10	169.3 (8)
S1—C1—C2—C3	−0.2 (10)	S1X—C1—C11—C10	−16.2 (3)
C1—C2—C3—C4	0.8 (13)	S1—C1—C11—C10	166.87 (13)
C2—C3—C4—S1	−1.1 (12)	C9—C10—C11—O1	−12.0 (2)
C2—C1—S1—C4	−0.3 (6)	C9—C10—C11—C1	169.19 (12)
C11—C1—S1—C4	176.5 (3)	C14—C9—C12—C13	−55.14 (16)
C3—C4—S1—C1	0.8 (8)	C10—C9—C12—C13	177.64 (12)
C11—C1—C2X—C3X	176.6 (11)	C6—C5—C13—O2	168.8 (4)
S1X—C1—C2X—C3X	1.5 (17)	C6X—C5—C13—O2	−9.2 (9)
C1—C2X—C3X—C4X	−2 (2)	S2X—C5—C13—O2	167.2 (3)
C2X—C3X—C4X—S1X	2 (2)	S2—C5—C13—O2	−8.30 (19)
C2X—C1—S1X—C4X	−0.2 (13)	C6—C5—C13—C12	−13.3 (5)
C11—C1—S1X—C4X	−175.4 (9)	C6X—C5—C13—C12	168.7 (9)
C3X—C4X—S1X—C1	−1.1 (19)	S2X—C5—C13—C12	−14.9 (4)
C13—C5—C6—C7	−178.8 (4)	S2—C5—C13—C12	169.61 (11)
S2—C5—C6—C7	−1.6 (7)	C9—C12—C13—O2	−38.13 (19)
C5—C6—C7—C8	1.4 (9)	C9—C12—C13—C5	143.98 (13)
C6—C7—C8—S2	−0.6 (9)	C10—C9—C14—C15	62.28 (18)
C7—C8—S2—C5	−0.3 (7)	C12—C9—C14—C15	−64.11 (18)
C6—C5—S2—C8	1.1 (5)	C10—C9—C14—C19	−114.87 (14)
C13—C5—S2—C8	178.7 (3)	C12—C9—C14—C19	118.75 (14)
C13—C5—C6X—C7X	174.6 (19)	C19—C14—C15—C16	1.2 (2)
S2X—C5—C6X—C7X	−2 (2)	C9—C14—C15—C16	−176.13 (13)
C5—C6X—C7X—C8X	−2 (4)	C19—C14—C15—Cl1	−179.44 (10)
C6X—C7X—C8X—S2X	6 (4)	C9—C14—C15—Cl1	3.24 (19)
C6X—C5—S2X—C8X	4.5 (13)	C14—C15—C16—C17	−1.1 (2)
C13—C5—S2X—C8X	−172.4 (9)	Cl1—C15—C16—C17	179.50 (12)
C7X—C8X—S2X—C5	−6 (2)	C15—C16—C17—C18	0.2 (2)
C14—C9—C10—C11	154.89 (12)	C16—C17—C18—C19	0.5 (2)
C12—C9—C10—C11	−76.57 (15)	C17—C18—C19—C14	−0.3 (2)
C2—C1—C11—O1	164.1 (6)	C17—C18—C19—Cl2	178.53 (12)
C2X—C1—C11—O1	−9.6 (8)	C15—C14—C19—C18	−0.5 (2)
S1X—C1—C11—O1	164.9 (3)	C9—C14—C19—C18	176.96 (13)
S1—C1—C11—O1	−12.0 (2)	C15—C14—C19—Cl2	−179.27 (10)
C2—C1—C11—C10	−17.0 (6)	C9—C14—C19—Cl2	−1.84 (18)

### 3-(2,6-Dichlorophenyl)-1,5-bis(thiophen-2-yl)pentane-1,5-dione Hydrogen-bond geometry (Å, º)

**Table:**

*D*—H···*A*	*D*—H	H···*A*	*D*···*A*	*D*—H···*A*
C3—H3···O2^i^	0.95	2.48	3.238 (8)	136
C9—H9···Cl2	1.00	2.47	3.0884 (18)	120
C9—H9···O1	1.00	2.44	2.819 (2)	102
C10—H10*A*···Cl1	0.99	2.75	3.3095 (19)	116
C10—H10*A*···S1*X*	0.99	2.73	3.085 (4)	102
C12—H12*A*···Cl1	0.99	2.64	3.317 (2)	126
C12—H12*A*···S2*X*	0.99	2.59	3.135 (6)	114
C4—H4···Cg5^ii^	0.95	2.83	3.431 (7)	122
C10—H10*B*···Cg1^ii^	0.99	2.72	3.704 (4)	171
C10—H10*B*···Cg3^ii^	0.99	2.74	3.720 (6)	170
C3*X*—H3*X*···Cg5^ii^	0.95	2.97	3.528 (14)	119
C8*X*—H8*X*···Cg2^iii^	0.95	2.91	3.699 (17)	141
C8*X*—H8*X*···Cg4^iii^	0.95	2.97	3.743 (18)	139

Symmetry codes: (i) *y*, −*x*+1/2, −*z*+1/2; (ii) −*x*+1, −*y*+1, −*z*; (iii) *x*−1/2, *y*−1/2, −*z*+1.
